# Smart Manufacturing and Production Flexibility under Market and Demand Fluctuations: Evidence from Electrical and Electronic Industrial Companies in Iraq

**DOI:** 10.12688/f1000research.176817.2

**Published:** 2026-05-26

**Authors:** Raafat A. Hussein, Nashwan M. Abdulaali, Shahla S. Khalil, Wijdan H. Hamoody

**Affiliations:** 1 Northern Technical University, Mosul, Nineveh Governorate, Iraq

**Keywords:** Smart Manufacturing, Production Flexibility, Industrial Companies

## Abstract

**Objective:**

To study and analyze the role of smart manufacturing technologies in enhancing the production flexibility of industrial enterprises by enabling rapid responses to market and demand fluctuations, minimizing downtime and operational waste, and improving efficiency in resource utilization, thereby contributing to sustainable competitive advantage.

**Research Design and Methods:**

The article is based on a survey. Data were collected through a survey conducted in electrical and electronic industrial companies in Iraq, which is considered a significant factor in its Production Flexibility. These companies were selected because they primarily implement smart manufacturing technologies and practices. Confirmatory factor analysis was used because it adopted the balanced free least squares method instead of the maximum likelihood method.

**Findings:**

The research results indicate that smart manufacturing represents a strategic approach enhances the ability of organizations to respond effectively to market fluctuations and demand levels, in addition The results showed that organizations that adopt smart manufacturing have greater organizational flexibility and productivity compared to traditional organizations, which positively impacts their ability to cope with uncertainty in the business environment.

**Implications and Recommendations:**

Industrial organizations are encouraged to invest in smart manufacturing technologies such as artificial intelligence, the Internet of Things, and predictive analytics to enhance production flexibility. In addition, organizations should focus on building human capabilities through continuous employee training and technological qualification programs to improve readiness for digital transformation.

**Contribution and Value Added:**

While previous studies have not quantified The Role of Smart Manufacturing in Supporting Production Flexibility in Light of Market and Demand Fluctuations in industrial companies, this article makes an important contribution by providing empirical evidence on how industrial companies view their policies as a useful tool in meeting their needs when providing support and assistance for the flexibility of its production processes in industrial companies.

## Introduction

In today’s industrial market environment, which is characterized by dynamic market conditions and persistent volatility in demand patterns, traditional production strategies can no longer accommodate rapid changes. Consequently, the ability to adapt and sustain production flexibility is emerging as an important force for business survival and competitive differentiation. In this scenario, smart manufacturing has become an area where a revolution has begun to take place, influencing the foundations of our industry.

Smart manufacturing is grounded in the integration of advanced technologies, such as artificial intelligence (AI), the Industrial Internet of Things (IIoT), and big data analytics, which collectively enable the creation of intelligent, interconnected, and adaptive production systems. This evolution extends beyond simple process automation toward the realization of “thinking factories” that are capable of learning from data, self-optimizing, and proactively responding to internal and external changes. Through such integration, smart manufacturing introduces a fundamental shift in industrial practice, enhancing both efficiency and flexibility, while empowering organizations to manage market volatility with greater agility ( Schlemitz & Mezhuyev, 2024a, 2024b).

This study explores the important role of smart manufacturing in increasing production flexibility. In particular, it analyzes how AI, IIoT, and big data analytics contribute to companies’ ability to adapt faster to changes in demand, increase operational efficiency, and reduce response times to market shifts. Moreover, the analysis focused on on-demand production and predictive maintenance, illustrating how these technologies contribute to building resilient, adaptive, and sustainable industrial systems capable of thriving amid ongoing economic and operational challenges.

This study had both primary and secondary objectives. The purpose is to underscore how important it is to increase production flexibility in an environment with market changes and unsteady demand; smart manufacturing is thus indeed a significant strategy for enhancing such flexibility. The aim of this undertaking is to explore how intelligent manufacturing technologies contribute to production flexibility in industrial settings. By doing so, we hope to boost the ability of these organizations to adapt to changing market demands, ultimately leading to sustainable competitive performance. This dual objective was the driving force behind this initiative. The goal of this study was to achieve the following goals aimed at carrying through:

1. To examine the relationship between smart manufacturing applications, such as artificial intelligence (AI), the Internet of Things (IoT), predictive analytics, and the levels of production flexibility within industrial organizations.
2. Analyze the impact of smart manufacturing on improving resource utilization efficiency and minimizing operational waste, thereby enhancing organizations’ responsiveness to market fluctuations.
3. To understand how smart manufacturing can increase the sustainability of supply chains and, at the same time, aid companies in responding to unexpected issues that could disrupt the production process.
4. To study ways in which smart manufacturing can improve product quality and make organizations more agile to meet different customer requirements efficiently and effectively.
5. To develop a pragmatic and conceptual basis, including smart manufacturing technologies and techniques, to enable the strategic deployment of smart manufacturing technologies to promote production flexibility and ensure sustainable competitive standing in a dynamic industrial environment.

The findings of this study are extremely significant because they contribute to the reduction of the knowledge gap that exists between the theories that underpin smart manufacturing technologies such as artificial intelligence, the Internet of Things, and predictive analytics, and their application in rendering production and operations more flexible. This study aims to contribute to the development of a body of scientific research that demonstrates the specific ways in which these technologies can assist with strategic decision-making, protect against shifts in demand, and ensure that supply chains last for a longer period of time, while maintaining high standards for product quality and operational efficiency.

Accordingly, this research seeks to address the following key questions:

1. What are the primary features of smart manufacturing that contribute to supporting production flexibility?
2. How can organizations effectively respond to fluctuations in the market and demand that are unpredictable through the use of smart manufacturing?
3. What challenges may arise in the implementation of smart manufacturing, and how can these challenges be addressed to achieve full production flexibility?

Smart manufacturing is an important new way of doing business that can help companies deal with the increasing difficulties of today’s business world, especially when the market is unstable and the demand changes. This makes this research very important. In this case, simply being efficient in production does not make you competitive anymore. Instead, a high level of production flexibility is necessary for companies that want to react quickly once the market changes.

The following sections of this document are organized as follows. First, a review of existing research is presented, followed by a discussion of the research findings. This paper concludes with a summary of the main points and suggestions for future research. The first section presents a literature review.

### Ethical considerations

This study involved human participants through a structured questionnaire distributed to employees and managers in industrial companies.

Ethical approval was not formally required for this study according to the institutional guidelines of Northern Technical University, as the research did not involve clinical experiments or sensitive personal data. However, the study was conducted in accordance with general ethical research principles.

All participants were informed about the purpose of the study, and their participation was entirely voluntary. The confidentiality and anonymity of all participants were strictly maintained, and no personally identifiable information was collect.

## Literature review

### Smart manufacturing

There have been a lot of “industrial revolutions,” the most recent of which was the Fourth Industrial Revolution, or Industry 4.0. All of these factors had a significant impact on the manufacturing sector. Several new technologies and ideas have emerged in the industrial era. These have been shown to make manufacturing easier, more efficient, and more competitive worldwide. These new ideas make smart manufacturing one of the most important ways to help the economic growth.

Linking machines through the Internet of Things (IoT) or other communication networks is not always used in smart manufacturing. This approach considers the entire industrial system. It connects different parts of an industrial ecosystem using data-based smart technologies and processes. This integration helps businesses get closer to being fully digital, which keeps them in touch with rapidly changing market conditions, shifting customer tastes, and growing environmental needs in real-time.

Smart manufacturing technologies help factories better use resources and make their operations more adaptable. This provides them with a long-term edge over their competitors. These tools help companies become accustomed to changes in the market more quickly, boost their ability to solve problems, and make the most out of new opportunities ( Wang et al., 2024; Zhang & Lee, 2025). In addition, smart manufacturing is better for the environment because it uses less energy and produces less waste, thus protecting resources for future generations ( Kumar et al., 2023; Li, Zhao, & Huang, 2025). Using IoT, AI-driven analytics, and cloud computing all at once makes it easier to make decisions immediately. This helps make the kind of production settings that can change and are strong, which is in line with the ideas of Industry 4.0 ( Smith & Chen, 2024; Garcia, et al., 2025).

Internet of Things (IoT), cyber-physical systems, and cloud computing are advanced technologies that have made smart manufacturing much more popular in today’s production environment. All of these technologies change the way things are made, increase the value of goods, and help different people ( Schlemitz & Mezhuyev, 2024, 135). According to Fan et al. (2025, 17), smart manufacturing environments have come into being to bring human and robotic systems closer together by combining different types of data and encouraging cooperation, which makes the system more productive, adaptable, and efficient. In addition, setting standards and making things work together are seen as very important for the success of smart manufacturing. They ensure that systems can work with each other, connect without a hitch, and run efficiently ( Villigran, et al., 2019, 352).

Ren et al. (2024, 17), however, pointed out that some problems still need to be addressed. Before smart manufacturing can be realized. Their assertion is that this is a situation. This is what they claim is a situation. The modeling and simulation of manufacturing equipment, processes, and workshops, which are activities that require a significant amount of human expertise and effort, are particularly susceptible to these challenges. These challenges are prevalent in the manufacturing industry. To achieve higher levels of efficiency, precision, and specialization, it is necessary to employ unconventional methods of thinking, rapid iteration, and intelligent monitoring mechanisms. This is because of the challenges presented. Similarly, Qu et al. (2019) pointed out that smart manufacturing relies on advanced intelligent systems that make it easier for products to be made quickly, for systems to respond quickly to changes, and for products, processes, distribution centers, and supply networks to be part of an interconnected industrial ecosystem. However, smart manufacturing cannot operate without these systems. These systems are necessary for smart manufacturing to be successful. As a result of this interconnectedness, the development of intelligent products and processes that can adjust to customer preferences and market dynamics is greatly encouraged. Smart manufacturing is a system that is fully integrated and collaborative, and it is able to react in real time to changes in the conditions of the factory, the activities of the supply chain, and the requirements of the customers, as ( Nimmala 2019, 4) emphasizes. In conclusion, smart manufacturing is a system that responds to changes in factory conditions.

**Recent developments in smart manufacturing**

Recent studies have emphasized that smart manufacturing is becoming a core strategic approach for improving industrial responsiveness, resilience, and operational efficiency in highly dynamic markets. Advanced technologies such as artificial intelligence, industrial Internet of Things (IoT), digital twins, cloud computing, and predictive analytics are increasingly integrated into manufacturing systems to support real-time decision-making and adaptive production processes.

According to Garcia et al. (2025), AI-driven analytics significantly improve production agility and operational adaptability by enabling manufacturers to predict disruptions and optimize workflows dynamically. Similarly, Li and Wang (2025) highlighted the growing role of digital twins in enhancing manufacturing flexibility through virtual simulation and real-time system monitoring. Chen et al. (2024) also emphasized that smart manufacturing contributes to sustainable industrial performance by reducing operational waste and improving energy efficiency.

Furthermore, recent Industry 4.0 research indicates that smart manufacturing systems enhance supply chain resilience and organizational responsiveness during periods of market uncertainty and fluctuating customer demand ( Wang et al., 2024; Zhang & Lee, 2025). These developments demonstrate that smart manufacturing is evolving beyond automation toward intelligent and self-optimizing industrial ecosystems capable of supporting long-term competitive advantage.

**Dimensions of smart manufacturing**

Several researchers have examined the foundations of smart manufacturing and viewed them as essential dimensions or drivers for implementing smart manufacturing in industrial organizations. Notable studies in this area include those by Kusiak (2017, 158), Qu et al. (2019, 130) and Schlemitz and Mezhuyev (2024, 138).

**Process and manufacturing technology**

New technologies and methods in manufacturing will help us make new, smarter products that can adapt to new situations. These tools make it easier to quickly adapt to changes in technology and the market. They also ensure that all parts of the operational process work together as a value chain. They encourage new ideas and competition by making things cheaper and more efficient. This maintains the lead in the field.

The concept of single-batch production is an important part of this change. Thus, when conditions change, manufacturers can quickly change how they do things by combining different ways of making things, such as computer numerical control (CNC), additive manufacturing, and automation. In this way, computer-aided design (CAD) files tell manufacturing systems about product needs, and the systems come up with a range of options for making things by themselves ( Lu et al., 2019, 39).

**Resource sharing**

Smart manufacturing uses the best methods to obtain high quality and flexibility at a low cost. In a factory, people and machines are connected to the outside world. A multilayer structure makes it easy for many people to work together at the horizontal level. In this setup, cyberspace has the same physical resources as factory floors. This allows groups to work together and share. This helps suppliers obtain things and services to the right place on time, either for free or at a low cost. Regardless of the location or location, smart manufacturing can connect customers and supply chains ( Huang, 2021).

**Data and networks**

Enterprise resource planning (ERP) systems help to make vertically integrated manufacturing processes more common. This is an important way to create more opportunities for improvement and automation in the industry. This integration helps people share information, start relevant operational procedures, and control the process, which makes the production hierarchy more aligned with itself at all levels. Smart manufacturing based on data depends on big datasets that contain many different kinds of data. These datasets are characterized by volume, variety, velocity, and variability. Artificial intelligence (AI), the Internet of Things (IoT), cloud computing, and other technologies that make it possible are at the heart of its construction. Owing to these factors, the entire production life cycle requires settings that can be scaled and support large data storage, fast processing, and AI-powered advanced analytics. Here, the focus of smart manufacturing is on making products based on the customer so that the market and consumer tastes can be met. This method was improved by self-organizing and open resource management, data-driven process control, real-time self-monitoring, and adaptive self-learning. Using both historical and real-time data can help businesses adapt to changing conditions quickly and easily. This may result in better efficiency in manufacturing, product performance, and overall competitiveness ( Tao et al., 2018, 190).

**Predictive engineering**

Smart manufacturing utilizes simulation models and virtual technologies to anticipate future changes, support intelligent reprogramming, predictive analytics, autonomous control, and re-engineering. This transforms organizations from merely reactive to proactive. Through analysis, monitoring, and autonomous control, digital models have been developed to study relevant phenomena, predict future outcomes, and incorporate both physical and virtual sensors within the industrial environment.

**Sustainability**

Intelligent manufacturing is a new way of making things that are more efficient, cheaper, and do ot use as much energy ( Nimmala, 2019, 7). These methods require more resources and energy. They also make the environment healthier and safer at work, and the economy more competitive ( Chen et al., 2024; Kumar & Verma, 2023). Smart manufacturing is a large, planned way of doing things that has many benefits. It uses new technologies, manages energy, and ensures that products can be traced, vertical integration, virtual simulations, automation, flexible production systems, and interconnected network strategies. Ideas from Industry 4.0 were used to make this method ( Kannan et al., 2023; Li & Wang, 2025; Garcia et al., 2025). It aims to make things easier, get emissions close to zero, lower the risk of accidents in manufacturing, and make long-term management easier.

### What is flexibility

It’s a fundamental dynamic capability that allows organaizations to respond to changing environments in a timely and efficient manner ( Pivorienė, 2015, 436) ( Kalogiannidis et al., 2022. 259) and It measures the ability to adapt to change and often has multiple dimensions that impact value jointly yet differently ( Alolayyana et al., 2022,1338). It has many dimensions; in our research, we take five dimensions.

### Dimensions of production flexibility

**Volume flexibility**

Volume flexibility is the ability of a production process to change the amount of product it produces when customer demand changes. With this ability, businesses can quickly and easily adapt to market ( Tran et al. 2025, 3; Kavaliauskiene et al. 2022, 846). Ewis et al. (2025, 1618) say that volume flexibility is the ability of a business to produce more or less while still being efficient and making money. In real life, volume flexibility means being able to make more or less than the planned amount. This allows businesses to adjust to sudden changes in customer demand without hurting their operations or financing. It is often measured as the percentage of the total unit cost that increases after production planning changes. A high fraction indicates that the volume can change more easily. This is because firms are more likely to increase their production capacity (which makes it less likely that output will be limited) when the costs of having that capacity are low compared with the costs of making things. Additionally, when costs are still changing at the update stage, firms are motivated to change the amount they produce based on the new demand forecast. Volume flexibility depends on many factors, such as the cost of the production process and the ease of changing supply contracts. Both affect how easily a company can increase or decrease production in response to market changes.

**Mix flexibility**

A flexibility mix allows a business to change how it makes things, so it can make many different kinds of products with many different types of specifications ( Tran et al., 2025, 3; Kavaliauskiene et al., 2022, 846). It shows how good a factory is at making many different parts and products and quickly changing them to meet the needs of different products. Al-Obaidy et al. (2023, 4) also stated that if a manufacturing process can be easily changed to fit different products, it can produce different types of products in the same production run without any problems.

Mix flexibility, also known as resource or product flexibility, is the ability to change the amount of one type of product into a different type. People usually look at the cost of changing one unit of capacity made for a certain product into a different one to figure this out.

If you can show that you can be more flexible with your mix, you might be able to obtain a better deal. In real life, many factors affect how easy it is to change a mix. For example, it might be difficult or expensive to change the tools used to create new products, and it also takes time to train workers on how to use different machines.

**Product flexibility**

A manufacturing system with product flexibility can add new parts to systems that are already in place without having to make many changes ( Zhang, et al., 2017, 4). An important example of product flexibility is component similarity, which stresses the importance of using the same parts in different types of products. Increasing the number of components that are similar to each other reduces inventory needs and resource use, and simplifies manufacturing. The following promotes two mix and size freedom ( Salvador et al., 2007, 1173). Tran et al. (2025 , 5) stated that product flexibility should include both new product and modification flexibility. Product flexibility includes development and people that help businesses proactively grow and change their product lines, as well as products with structures and designs that can be changed. From these points of view, we can see how important it is for a company to be able to both make brand-new products and make small changes to things that they already sell. This ensures that they can adapt to market changes and new technologies.

**Resource flexibility**

Corporations tend to be more successful when they have further versus where they can obtain what they need. A supplier will be okay if they do not make enough of the things needed to make something or if the things they make are bad. The business has “supply flexibility” ( Ewis et al., 2025, 1617), which means it can choose a different supplier. Being resource flexible also means that one can handle materials in different ways, such as having and using machines to make production faster. A flexible workforce can quickly and cheaply complete a range of manufacturing jobs. If companies are flexible with their workers, they can quickly deal with unexpected increases in work ( Hatmanto & Yuniarinto, 2022, 3).

**Scheduling flexibility**

It is necessary to investigate a company’s ability to modify delivery routes, timelines, and schedules in response to customer requirements and unanticipated events when evaluating an organization’s capacity to deliver and adapt schedules. This is due to the fact that the evaluation is being conducted in relation to the organization’s capability of offering and adjusting schedules. Al-Obaidy et al. (2023, 4) defined scheduling or routing flexibility as “the capacity of a manufacturing system to generate products via diverse routes.” This definition applies to both scheduling and routing flexibilities. This description is applicable to two aspects of adaptability: scheduling and routing.

“Volume flexibility” comes from more general literature on operational flexibility. This means an organization’s ability to change its overall production or service levels to meet demand that is either going up or down while keeping the business running smoothly. With this kind of flexibility in operational management, you can quickly respond to changes in the market and customer needs. Along the same lines, mix flexibility shows how well an organization can change the types of products it delivers to the market, while still keeping costs low.

Importantly, mix flexibility depends on process and product flexibility. When customers need something, process flexibility refers to how quickly and easily a company can decide to do something, change their plans, or change an order that has already been placed. Product flexibility refers to how many companies can change products to meet customer needs. When demand changes significantly, product flexibility usually decreases. When products are strategic complements or substitutes, volume flexibility may increase or decrease, respectively. In addition, volume elasticity is better at making aggregate demand less uncertain, whereas product elasticity focuses on making individual product demand less uncertain.

### Research gap

Despite the growing body of literature on smart manufacturing and Industry 4.0 technologies, several important gaps remain insufficiently addressed. First, most previous studies have focused primarily on developed economies and large-scale manufacturing environments, while limited attention has been given to developing countries and emerging industrial contexts such as Iraq. Second, existing research has often examined smart manufacturing technologies from a technological or operational perspective without comprehensively analyzing their direct role in enhancing production flexibility under unstable market and demand conditions.

Moreover, previous studies rarely integrated the dimensions of smart manufacturing, including process technology, predictive engineering, sustainability, data and networks, and resource sharing, with multiple dimensions of production flexibility within a unified analytical framework. In addition, limited empirical evidence exists regarding how industrial organizations can utilize smart manufacturing technologies to improve responsiveness, adaptability, and operational resilience during periods of uncertainty and fluctuating customer demand.

Therefore, this study seeks to fill these theoretical and empirical gaps by providing an integrated analytical model that examines the relationship between smart manufacturing dimensions and production flexibility in electrical and electronic industrial companies in Iraq.

## Theoretical relationship

Modern literature increasingly recognizes that smart manufacturing is no longer merely a technological advancement within production environments; rather, it has evolved into a comprehensive organizational approach aimed at enhancing the adaptability and responsiveness of production systems in the face of continuous market fluctuations. Zheng *et al*. (2018) described smart manufacturing as a digital architecture in which integrated data and control systems support real-time decision-making, whereas Koren *et al*. (2018) emphasized reconfigurable systems as the structural foundation for achieving dynamic production flexibility, enabling rapid and cost-effective transformation of production lines.

Moreover, Wang and Gao (2020) highlighted that volatile economic conditions necessitate the adoption of flexible and resilient production systems that can be realized through the integration of artificial intelligence (AI) and the Internet of Things (IoT) for process monitoring and self-optimization. Xu *et al*. (2021) further noted the transformative role of the Fourth Industrial Revolution, which shifted production from static lines to learning systems enabled by digital twins and simulations. In this context, Mourtzis (2022) illustrates how digital twins function as testing platforms, allowing organizations to simulate and modify production scenarios before actual implementation.

Therefore, the relationship between intelligent manufacturing and production flexibility is neither linear nor purely mechanical; rather, it is dynamic and integrated. This is because smart manufacturing is a result of this problem. The power of a system to learn, change, and rebuild itself to meet the ever-changing demands of the market reflects where this integration is reflected. Therefore, flexibility is not merely a design feature, but rather a direct consequence of the intelligence that the system possesses.

## Research methodology

### Methodology of the investigation

This study employs a descriptive analytical framework. The theoretical component was elucidated through a descriptive method, whereas the analytical method was applied practically. This methodological design facilitated the testing of the study’s hypotheses by scrutinizing the interrelationships between the primary and secondary variables, drawing upon data acquired from the participating companies.

Hypothesis 1: A strong link exists between the overall aspects of smart manufacturing and production freedom.

The subsequent sub-hypothesis emerges from this premise:

A strong correlation is observed between smart manufacturing and diverse dimensions of production flexibility, including Volume Flexibility, Mix Flexibility, Product Flexibility, Process Flexibility, and Scheduling Flexibility.

Hypothesis 2 suggests that smart manufacturing significantly affects the overall production flexibility within the specific organizational setting being studied. Consequently, the following sub hypotheses are proposed:

Smart manufacturing significantly influences individual aspects of production flexibility, namely Volume Flexibility, Mix Flexibility, Product Flexibility, Process Flexibility, and Scheduling Flexibility.

### Informed consent

Informed consent was obtained from all participants prior to their participation in the study. Participants were clearly informed about the purpose of the research and their right to withdraw at any time without any consequences.

Consent was obtained verbally due to the nature of the data collection, as the questionnaire was distributed in field settings within industrial companies where written consent was not practical. Participation in the survey was considered as consent after explanation of the study objectives.

## Result and discussion

**First**, a description of the sample used for the research was provided. A purposive sample was selected, which included people who were familiar with the activities and tasks that were performed in the laboratory, as well as those who possessed experience and knowledge. This ensured that the information they provided was employed in a manner that was both accurate and helpful, and it also provided the opportunity to gather ideas and suggestions that would boost the significance of the research. In accordance with this, the researchers sent 240 questionnaires to the organization’s general manager, department heads, branch managers, unit and division managers, and production line supervisors. The total number of valid questionnaires collected for the purpose of analysis was 210, representing a response rate of 88%.

Confirmatory Factor Analysis (CFA) was employed as the second analytical approach in this study to evaluate the validity and reliability of the proposed measurement model. This method provides a set of goodness-of-fit indicators that determine the extent to which the observed data fit the theoretical model and support the testing of the research hypotheses. In the current study, the Balanced Free Least Squares (BFLS) estimation method was adopted instead of the Maximum Likelihood (ML) method in conducting CFA. This methodological choice was made because several assumptions required for the ML method, particularly multivariate normality and ideal sample adequacy conditions, were not fully satisfied by the collected data.

Previous methodological studies indicate that BFLS is more appropriate when data distributions deviate from normality or when sample characteristics may affect the robustness of covariance-based estimation methods. Therefore, the BFLS method was considered more suitable for achieving stable parameter estimation and improving the reliability of the measurement model under the current research conditions.

**Figure 1.**
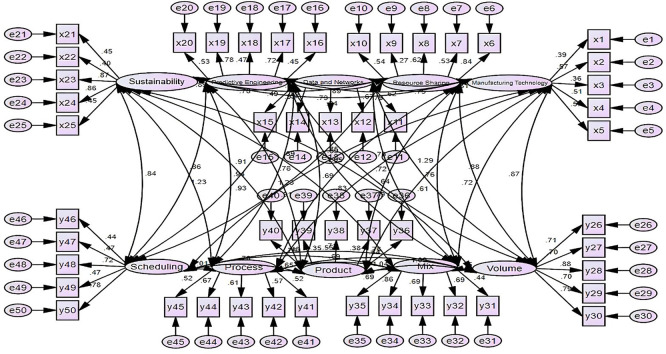
Conceptual framework illustrating the proposed relationships between smart manufacturing dimensions and production flexibility, highlighting the hypothesized direct and indirect effects.

Furthermore, the adopted estimation approach contributed to improving the validity of the structural model and ensuring acceptable goodness-of-fit indicators for testing the proposed research hypotheses, as illustrated in Figure 1 and Table 1.

**Table 1.** Research prototype quality indicators.

Standard indicators	Acceptance limits	Model indicators	Matching result
GFI **Goodness of Fit Index**	0.90 > GFI Model quality	0.926	Matching
AGFI **Adjusted Goodness of Fit Index**	0.90 > AGFI Best Match	0.916	Matching
RMR Root Mean Square Residuals	RMR value between 0.08 and zero	0.071	Matching
NFI Normed Fit Index	0.90 > NFI Best Match	0.913	Matching
RFI Relative **Fit** Index	0.90 < (RFI) Data fit to model	0.906	Matching
PGFI Parsimony Goodness of Fit Index	The closer to (1), the better the match.	0.820	Matching
PRATIO Practically Simple Model Index	PRATIO >0.90 Best Match	0.922	Matching

**Source**: Table prepared by researchers based on the results of the computer.

Based on the data shown in Table 1, the indicators of the hypothetical diagram are considered adequate, in addition to being within the confines of the model’s quality indicators. Therefore, the model is accepted without any adjustments, and it satisfies the prerequisites for moving on to the next step, testing the research hypothesis.

Confirmatory factor analysis was completed in conjunction to ensure that our research model is in line with the field data and meets the standards for the quality of matches. We will now proceed to this step.

**Figure 2.**
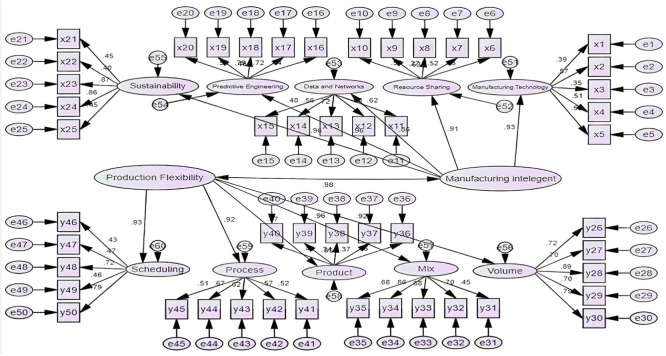
Confirmatory factor analysis (CFA) model showing the standardized factor loadings and the measurement structure of smart manufacturing and production flexibility constructs.

**The first major assumption** is that production flexibility is significantly correlated with the Dimensions of Smart Manufacturing (together), which is responsible for confirming the first main hypothesis that states that the Smart Manufacturing dimensions (together) and production flexibility are significantly correlated. The data in Table 2 show that this is the case, with a correlation coefficient of 0.979*, which is statistically significant at the level of 0.05. Therefore, the first hypothesis was confirmed. As shown in Table 3, the results demonstrate a strong and statistically significant relationship between smart manufacturing dimensions and production flexibility. This conclusion is further supported by Figure 2.

**Table 2.** The relationship between the of Smart Manufacturing dimensions (Combined) and production flexibility.

Independent variable\Dependent variable	Dimension Intelligent Manufacturing
**Production Flexibility**	0.979*

**Source**: Table prepared by researchers based on the results of the computer.

**Table 3.** Correlations between Smart Manufacturing and dimensions of production flexibility (individually).

Dimensions of production flexibility	Scheduling Flexibility	Process Flexibility	Product Flexibility	Mix Flexibility	Volume Flexibility
Intelligent manufacturing	0.998*	0.995*	0.907*	0.864*	0.837*

**Source**: Table prepared by researchers based on the results of the computer.

### The first hypothesis generates the following sub-hypothesis

**Figure 3.**
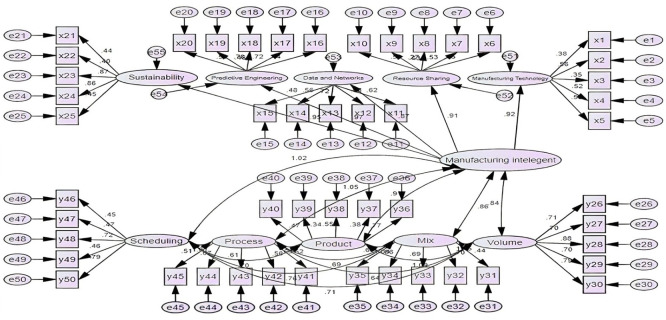
Structural equation model (SEM) illustrating the direct effect of smart manufacturing dimensions on overall production flexibility at the organizational level.

A notable correlation was observed between Smart Manufacturing and the individual dimensions of production flexibility, as evidenced by the following:

The smart manufacturing and production flexibility dimensions are related. The research results show that there is a strong link between smart manufacturing techniques and different types of production flexibility. As for volume adaptability, the results show a significantly positive association at a significance level of 0.05, with a correlation value of 0.837. This means that firms can better adjust the amount of production they do when demands changes when they use smart manufacturing systems. In the same way, the research shows that there is a strong link between smart manufacturing and mix flexibility. This can be seen through a correlation value of 0.864 at 0.05, which suggests that the ability to easily handle a range of products has been improved. There was a strong positive association (0.907, p < 0.05) in terms of product flexibility, which shows that using smart production technologies can help make products more adaptable and customizable. The link between smart manufacturing and process flexibility seems to be very strong, as shown by a correlation value of 0.995 at the 0.05 significance level. This shows that smart systems are very good at making it possible for quick changes to be made to processes. Finally, a correlation value of 0.998 (p < 0.05) shows a strong link between flexible scheduling and the use of smart manufacturing. This shows that production planning and task scheduling can be more sensitive, supporting hypothesis (A) of the first primary hypothesis, as Figure 3 shows a strong positive association between Smart Manufacturing and production flexibility.

### The second hypothesis

**Figure 4.**
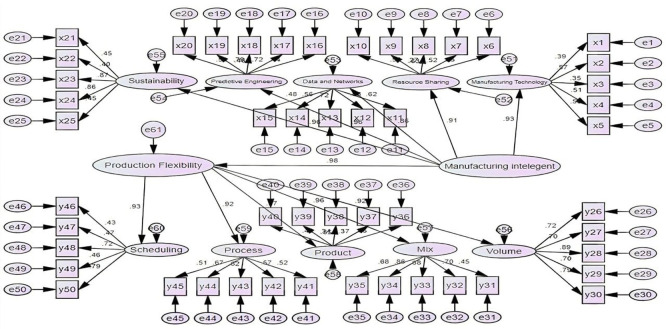
Structural equation model showing the effects of smart manufacturing dimensions on the individual dimensions of production flexibility, including volume, mix, product, process, and scheduling flexibility.

Smart Manufacturing has a considerable impact on the overall production flexibility at the organizational level. A structural equation model was constructed to test this hypothesis ( Figure 4). The test values of this model are presented, which indicate the acceptance or rejection of our hypothesis, as detailed in Table 4, as follows:

**Table 4.** Values of the second hypothesis analysis.

Influencing variable	Direction of effect	Variable affected by	Estimate	SRW	Upper	Lower	P
Manufacturing Intelligent	▼	Production Flexibility	2.232	0.979	1.001	0.954	0.04

**Source**: Table prepared by researchers based on the results of the computer.

**Figure 5.**
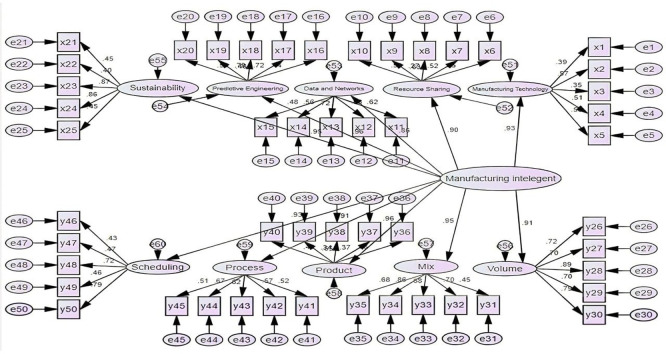
Path diagram presenting standardized regression coefficients and critical ratios for testing the research hypotheses related to smart manufacturing and production flexibility.

The data presented in Table 4 demonstrate a direct and significant effect of Smart Manufacturing on production flexibility, evidenced by a standard regression coefficient (SRW) of (0.979) and a non-standard regression coefficient (estimate) of (2.232). A P-value of 0.02, which is below the threshold of 0.05, indicates that the effect is significant. The data show the confidence range for nonstandard regression statistics. The confidence level was set to 95%. Zero was not in the range of 1.001–0.954. This clarifies how important it is to know how the explanatory variable changes; therefore, this study looks at the second main part, which is based on the dependent variable. We now examine the impact of smart manufacturing on the different types of production flexibility at the company level. To determine how this relationship works in real life, a theory was made that smart manufacturing has a statistically significant effect on all aspects of the organization’s ability to be flexible with production that is being studied. A method called Structural equation modeling (SEM) was used to determine whether this idea worked. Figure 5 shows the suggested model and Table 5 shows the data and model fit results. These results indicate that the suggested idea is correct or incorrect.

**Table 5.** Values of the second hypothesis analysis.

Influencing variable	Direction of effect	Variable affected by	Estimate	SRW	Upper	Lower	P
Smart Manufacturing	▼	Volume Flexibility	2.083	0.911	0.967	0.821	0.03
Smart Manufacturing	▼	Mix Flexibility	1.000	0.950	1.002	0.864	0.04
Smart Manufacturing	▼	Product Flexibility	1.403	0.964	1.058	0.874	0.01
Smart Manufacturing	▼	Process Flexibility	1.762	0.914	0.998	0.822	0.03
Smart Manufacturing	▼	Scheduling Flexibility	2.278	0.927	0.980	0.870	0.04

**Source**: Table prepared by researchers based on the results of the computer.

Structural equation modeling showed that the model created to test sub-hypothesis (A) of the second main hypothesis is important. This is clear from the positive signs in Table 5 and the high saturation values over 45%, as shown in Figure 5. By examining the typical error values, it becomes clear that the highest impact of the dimensions of Intelligent Manufacturing (Combined) was in the dimension (Product Flexibility), which indicates that the organization under study seeks to maintain the production of flexible products that meet the needs and desires of customers by adopting a series of operations through which waste is recycled and used in remanufacturing again, while the lowest impact of the dimensions of Smart Manufacturing (Combined) was in the dimension (Product Flexibility), as the organization lives in a turbulent, rapidly changing environment that is unable to predict what is coming as a result of the surrounding changes in an external environment, The results show that the critical ratio (C.R.) value of 4.71 is higher than the 1.96 and 2.66 threshold values at the 0.05 and 0.01 levels, respectively. These were the same as the t-values used in the standard regression analysis. This result proves that the expected effects were statistically significant. Thus, the second sub-hypothesis is proven to be true. This shows that intelligent manufacturing dimensions working together have a significant effect on the single dimensions of production freedom.

### Challenges and implementation barriers

Despite the significant advantages of smart manufacturing in enhancing production flexibility, several implementation challenges remain critical for industrial organizations. One of the primary barriers is the high initial investment cost associated with adopting advanced technologies such as artificial intelligence, industrial Internet of Things (IoT), cloud computing, and digital twin systems. Many industrial organizations, particularly in developing economies, may face financial limitations that restrict large-scale digital transformation initiatives.

Another major challenge relates to workforce readiness and technical expertise. Successful implementation of smart manufacturing systems requires skilled employees capable of operating, analyzing, and maintaining advanced digital technologies. Therefore, continuous employee training and organizational learning are essential to ensure effective technology adoption and operational sustainability.

Cybersecurity risks also represent an important concern in smart manufacturing environments due to the increasing interconnectivity between machines, data systems, and industrial networks. Unauthorized access, data breaches, and system vulnerabilities may negatively affect operational reliability and organizational performance.

Furthermore, integrating smart manufacturing technologies into existing industrial infrastructures can be technically complex and may require gradual implementation strategies, organizational restructuring, and continuous technological adaptation. Addressing these challenges is essential for achieving long-term operational flexibility and sustainable competitive performance.

## Conclusions

By doing this study, we tried to determine whether, and to what degree, the dimensions of smart manufacturing promote the increased production flexibility of industrial firms. Aligning with our hypotheses, our results demonstrate that firms that integrate technologies and processes for smart manufacturing are expected to perform more operational resilience. Thus, we also conclude that this research addresses from an empirical perspective how firms employ smart manufacturing to ensure flexible production processes to achieve organizational goals and the common public, in the wider society of the regions where they work, thereby helping their communities, and have contributed to the process of the value chain. Educationally, it is important to study how business managers develop and function in the contribution and importance of smart manufacturing dimensions to production flexibility. According to the research gap, there is a clear gap in the current literature on the nature of production flexibility, especially on smart manufacturing dimensions and technologies, industrial manufacturing. Our results further suggest that maintaining and building relationships with key stakeholders is key to successful initiatives, such as sustainable initiatives, a quintessential dimension of smart manufacturing. Thus, business managers can strategically plan to increase the frequency and quality of communication in their networks, while maintaining stakeholder engagement to serve operational goals as well as community-based goals. These findings are as follows. The study revealed from the applied side of this study that smart manufacturing acts as a strategic technique for industrial agencies to increase the flexibility of manufacturing and, accordingly, strengthen the capacity to respond to market changes and fluctuations and fluctuating demand levels. The outcome of this study showed that smart manufacturing dimensions can contribute directly to improving the efficiency of resource utilization and minimizing negative operational waste through the integration of smart manufacturing dimensions in process and manufacturing technology, data and networks, predictive engineering, sustainability (for processes), and resource sharing. The findings established that smart manufacturing improves production agility through digitization and instant data transfer, thus reducing external disturbances to the production flow. Smart manufacturing is not only advantageous with regard to products but also a quick response towards fluctuating customer demand for customer satisfaction and organizational competitiveness. This is impressive for organizations adopting smart manufacturing, as it allows for more organizational flexibility and productivity, which helps companies manage ambiguity in dynamic business settings. Finally, this study demonstrated that it is possible to generate a theoretical and practical framework that describes how to utilize smart manufacturing to generate an ideal trade-off between efficiency and flexibility for long-term sustainable competitive performance in a fast-competitive world.

### Limitations

This study was limited to electrical and electronic industrial companies in Iraq, which may affect the generalizability of the findings to other industrial sectors or countries with different technological and economic conditions. In addition, the study adopted a cross-sectional survey design, which limits the ability to examine long-term changes and dynamic developments in smart manufacturing implementation over time. Furthermore, some statistical assumptions related to multivariate normality and sample adequacy were not fully satisfied, which led to the adoption of the Balanced Free Least Squares estimation method.

### Future research directions

Future studies are encouraged to investigate smart manufacturing and production flexibility across different industrial sectors and geographical contexts to improve the generalizability of findings. Longitudinal research designs are also recommended to evaluate the long-term sustainability and adaptability of smart manufacturing systems under rapidly changing market conditions. In addition, future research may explore the impact of cybersecurity readiness, organizational culture, digital transformation maturity, and employee technological capabilities on the successful implementation of smart manufacturing systems.

## Data Availability

Zenodo: Survey Data for Smart Manufacturing and Production Flexibility. https://doi.org/10.5281/zenodo.19201585 [ Aloubady, rafat et al. (2026)]. The project contains the following underlying data: Data Research.csv (raw survey data exported from SPSS). Questionnaire_Smart_Manufacturing.docx (original questionnaire used in the study). Zenodo: Survey Data for Smart Manufacturing and Production Flexibility. https://doi.org/10.5281/zenodo.19201585 [ Aloubady, rafat et al. (2026)]. This project contains the following extended data: Data Research.sav (original SPSS dataset file). Data are available under the terms of the Creative Commons Zero (CC0 1.0 Public domain dedication).
