# Supplementary material for: Smart Manufacturing and Production Flexibility under Market and Demand Fluctuations: Evidence from Electrical and Electronic Industrial Companies in Iraq
Source: F1000Res. 2026 May 26;15:587. Originally published 2026 Apr 19. [Version 2] doi: 10.12688/f1000research.176817.2 (PMC13247568; doi:10.12688/f1000research.176817.2)
Supplement: Peer review history

# Reviewer report: Reviewer response for version 2

**DOI:** 10.5256/f1000research.201282.r488542

**Published:** 2026-06-08

**Authors:** Attia Hussien Gomaa

**Affiliations:** 1 Benha University, Banha, Al Qalyubia Governorate, Egypt

Recommendation: Accept Submission

 After careful review of the manuscript, I recommend accepting this manuscript.

Is the work clearly and accurately presented and does it cite the current literature?

Partly

If applicable, is the statistical analysis and its interpretation appropriate?

Partly

Are all the source data underlying the results available to ensure full reproducibility?

Partly

Is the study design appropriate and is the work technically sound?

Partly

Are the conclusions drawn adequately supported by the results?

Partly

Are sufficient details of methods and analysis provided to allow replication by others?

Partly

Reviewer Expertise:

Smart Manufacturing

I confirm that I have read this submission and believe that I have an appropriate level of expertise to confirm that it is of an acceptable scientific standard.

# Reviewer report: Reviewer response for version 2

**DOI:** 10.5256/f1000research.201282.r488543

**Published:** 2026-06-05

**Authors:** Bikash Koli Dey

**Affiliations:** 1 SRM Institute of Science and Technology, Kattankulathur, Tamilnadu, India

The present form of the manuscript may be accepted for indexing

Is the work clearly and accurately presented and does it cite the current literature?

Yes

If applicable, is the statistical analysis and its interpretation appropriate?

No

Are all the source data underlying the results available to ensure full reproducibility?

Yes

Is the study design appropriate and is the work technically sound?

Yes

Are the conclusions drawn adequately supported by the results?

Partly

Are sufficient details of methods and analysis provided to allow replication by others?

Yes

Reviewer Expertise:

Smart Manufacturing, Intelligent manufacturing, Supply chain management, Inventory control

I confirm that I have read this submission and believe that I have an appropriate level of expertise to confirm that it is of an acceptable scientific standard.

# Reviewer report: Reviewer response for version 1

**DOI:** 10.5256/f1000research.194924.r477383

**Published:** 2026-05-06

**Authors:** Bikash Koli Dey

**Affiliations:** 1 SRM Institute of Science and Technology, Kattankulathur, Tamilnadu, India

This research digs into how smart manufacturing tech helps industries stay flexible when markets and demand swing around. It focuses on electrical and electronic companies in Iraq that use these smart basics to boost their production adaptability. Although the study presents valuable insights into the role of smart manufacturing in enhancing production flexibility, several critical issues merit attention. Firstly, the research relies heavily on a survey approach focused solely on electrical and electronic industries in Iraq, which may limit the generalizability of findings to other sectors or regions with different technological maturity levels. The methodology utilizes confirmatory factor analysis with a balanced free least squares method, yet the paper notes that necessary conditions such as data normality and sufficient sample size were not fully met, potentially impacting the robustness of the statistical results. Additionally, while the study demonstrates significant correlations between smart manufacturing and production flexibility dimensions, it does not deeply explore challenges or barriers to implementation, such as costs, workforce readiness, or cybersecurity risks associated with advanced technologies like AI and IoT. The authors also mention the complexity and expertise required for modeling and simulation but provide limited practical guidelines or strategies to overcome these obstacles. Furthermore, questions remain about the long-term sustainability and adaptation of smart manufacturing systems in rapidly evolving markets, especially given the unclear impact on product flexibility. Future research should thus address these gaps, employing more diverse samples and longitudinal designs to validate and extend the empirical findings.

Is the work clearly and accurately presented and does it cite the current literature?

Yes

If applicable, is the statistical analysis and its interpretation appropriate?

No

Are all the source data underlying the results available to ensure full reproducibility?

Yes

Is the study design appropriate and is the work technically sound?

Yes

Are the conclusions drawn adequately supported by the results?

Partly

Are sufficient details of methods and analysis provided to allow replication by others?

Yes

Reviewer Expertise:

Smart Manufacturing, Intelligent manufacturing, Supply chain management, Inventory control

I confirm that I have read this submission and believe that I have an appropriate level of expertise to confirm that it is of an acceptable scientific standard, however I have significant reservations, as outlined above.

# Reviewer report: Reviewer response for version 1

**DOI:** 10.5256/f1000research.194924.r477390

**Published:** 2026-05-05

**Authors:** Attia Hussien Gomaa

**Affiliations:** 1 Benha University, Banha, Al Qalyubia Governorate, Egypt

Thank you for submitting your manuscript. Your study addresses a timely and significant topic with strong potential to advance the field. However, the manuscript requires substantial revision to meet the journal’s standards for rigor, clarity, and scholarly impact.

 Please address the following points carefully in your revised submission. Highlight all changes in the manuscript and provide a point-by-point response for each comment.

**Main Concerns:**

**1. Title: **The title appears partially misaligned with the study’s objectives and analytical scope. It should be refined to more accurately reflect the research focus, context, and methodological orientation.

**2. Literature Review: **The literature review is largely descriptive and lacks sufficient critical synthesis. It should incorporate more recent high-impact studies (preferably 2023–2026) and strengthen its theoretical foundation. The section should better position the study within the current literature and more strongly integrate AI-driven marketing and digital transformation research.

**3. Challenges and Research Gaps Analysis: **A dedicated section is recommended to identify theoretical, empirical, and methodological gaps explicitly. This would strengthen the justification of the study’s originality and clarify its contribution.

**4. Methodology: **The methodology requires a clearer and more systematic presentation. Key procedures should be explicitly justified and transparently explained. The use of conceptual frameworks, diagrams, or flowcharts is strongly recommended to enhance clarity and reproducibility.

**5. Results and Discussion: **The results should be presented in a structured manner with clear alignment to the research questions. The discussion should move beyond description to provide deeper interpretation, stronger engagement with relevant literature, and clearer articulation of theoretical and practical implications.

**6. Conclusion, Limitations, and Future Research: **These sections require refinement for improved clarity and coherence. The conclusion should better synthesize the main findings and contributions, while limitations and future research directions should be explicitly grounded in the study’s scope and identified gaps.

**7. References and Citation Consistency: **The reference list contains inconsistencies, including incomplete entries and missing bibliographic details. There is also a mismatch between in-text citations and the reference list. Additionally, reliance on non-academic sources should be minimized and supplemented with peer-reviewed, high-impact scholarly literature.

**8. Examples of References Requiring Revision**

1. Aloubady, R. M., Nashwan Mohammed, A., Shahla, S. K., et al. (2026). Survey data for smart manufacturing and production flexibility [Data set]. Zenodo.
2. Huang, Z. (2021). *Smart manufacturing based on dynamic value stream mapping* (PhD thesis). University of Queensland.
3. Izadkhah, A., Subramanyam, A., Lainez-Aguirre, J. M., et al. (2022). Delivery day flexibility and last-mile costs. *Digital Chemical Engineering*, 5, 100057.
4. Lu, Y., Huang, H., Liu, C., et al. (2019). Standards for smart manufacturing. In *IEEE CASE*.
5. Tao, F., Qi, Q., Liu, A., et al. (2018). Data-driven smart manufacturing. *Journal of Manufacturing Systems*, 48, 157–169.
6. Tran, T. T. H., Van den Broeke, M., & Paparoidamis, N. G. (2025). Flexibility and firm performance. *Journal of Business Research*, 194, 115314.
7. Wang, L., & Gao, R. X. (2020). Flexible and resilient production systems. *Journal of Manufacturing Systems*, 56, 157–169.

**9. Writing Quality: **The manuscript requires improvement in academic writing quality, particularly in clarity, coherence, terminology consistency, and logical flow of arguments.

**Overall Assessment: **The manuscript presents a promising research direction but requires revision and refinement before it can be indexed. Key weaknesses relate to literature integration, structural completeness, methodological clarity, reference integrity, and academic writing quality.

Is the work clearly and accurately presented and does it cite the current literature?

Partly

If applicable, is the statistical analysis and its interpretation appropriate?

Partly

Are all the source data underlying the results available to ensure full reproducibility?

Partly

Is the study design appropriate and is the work technically sound?

Partly

Are the conclusions drawn adequately supported by the results?

Partly

Are sufficient details of methods and analysis provided to allow replication by others?

Partly

Reviewer Expertise:

Smart Manufacturing

I confirm that I have read this submission and believe that I have an appropriate level of expertise to confirm that it is of an acceptable scientific standard, however I have significant reservations, as outlined above.
